# Improving Child Survival in Sub-Saharan Africa: Key Environmental and Nutritional Interventions

**DOI:** 10.5334/aogh.2908

**Published:** 2020-07-06

**Authors:** A. Kofi Amegah

**Affiliations:** 1 Public Health Research Group, Department of Biomedical Sciences, University of Cape Coast, Cape Coast, GH

## Abstract

Many countries in Sub-Saharan Africa (SSA), did not achieve the Millennium Development Goal 4 target of reducing under-five mortality by two-thirds between 1990 and 2015. A large proportion of under-five deaths in SSA and other developing regions have been attributed to undernutrition and poor household environmental conditions. Failure to address nutritional deficit and household environmental pollution in SSA will therefore likely result in many countries not meeting the Sustainable Development Goal (SDG) 3.2 target which aims to reduce under-five mortality to less than 25 deaths per 1000 livebirths by 2030. This paper pinpoints the nutritional and environmental threats to child health in SSA, and identify interventions that will work best to improve child survival in countries. It is important to broaden the spectrum of interventions for improving child survival beyond health systems strengthening to enable countries meet the SDG 3.2 target. The following interventions are thus proposed: strengthening child welfare clinics through digital technologies; investment in school feeding programmes; addressing household air pollution; and improving water, sanitation and hygiene (WASH) services in basic schools. There are certainly barriers to effective implementation of the proposed interventions in countries but are surmountable with strong political will and involvement of the private sector.

## Introduction

Gains in child survival have long being recognized as an important proxy indicator of improvements in overall population health and socioeconomic development [1]. Several countries worldwide have recorded substantial reductions in under-five mortality over the past three decades [2]. Many countries in Sub-Saharan Africa (SSA), however, did not achieve the Millennium Development Goal (MDG) 4 target of reducing under-five mortality by two-thirds between 1990 and 2015 [2]. According to the 2015 Global Burden of Disease (GBD) estimates, only two countries, Ethiopia and Liberia, achieved the MDG4 target [3]. Also, many of the 23 countries globally reported as having under-five mortality rates of 75+ deaths per 1000 livebirths in 2015 were found in SSA [3]. These rates are three times higher than the Sustainable Development Goal (SDG) target 3.2 which aims to reduce under-five mortality to <25 deaths per 1000 livebirths by 2030. The annualized rate of decline of under-five mortality was also low in many SSA countries [3]. An assessment of under-five mortality in 99 low- and middle-income countries and across 46 African countries also noted significant within-country heterogeneity in SSA [4,5]. Burstein et al. also observed under-five mortality rates of ≥80 deaths per 1000 livebirths across large geographical areas in Western and Central SSA [4].

A large proportion of under-five deaths in SSA and other developing regions have been attributed to undernutrition and poor household environmental conditions. Childhood illnesses with high mortality burden such as malaria, gastroenteritis, pneumonia and acute lower respiratory infections are all linked to household environmental exposures [6]. Undernutrition and environmental infections are inextricably linked. However, according to the World Bank, over time, these links have been neglected by policy makers in their formulation of strategies for improving child survival [7].

Failure to address nutritional deficit and household environmental pollution in SSA will thus likely result in many countries not meeting the SDG 3.2 target. It is against this background that this paper pinpoints the nutritional and environmental threats to child health in SSA, and identify interventions that will work best to improve child survival in countries. In many SSA countries interventions for improving child survival has focused predominantly on health systems strengthening and it is important to broaden the spectrum of interventions if countries are to meet the SDG 3.2 target.

## Nutritional and Environmental Threats to Child Survival

Growth monitoring assures early detection of growth faltering, improved nutrition, and reduction in childhood morbidity and mortality. A review found children who undergo growth monitoring coupled with their mothers receiving nutrition and health education as part of the process to have better nutritional status and/or survival rate [8]. The findings were independent of immunization and socioeconomic status.

Breastfeeding confers survival throughout the entire continuum of childhood. Optimal breastfeeding prevents around 12% under-five deaths annually in low- and middle-income countries [9]. All-cause and infection-related mortality in infants and children have been reported to be higher in partially- and non-breastfed infants compared to exclusively breastfed infants [10]. Early initiation of breastfeeding is associated with reduced risk of neonatal mortality [11]. Exclusive breastfeeding also confers a lower risk of mortality including infection-related deaths in the neonatal period [11].

Complementary feeding is important for child growth and development. Provision of complementary foods with and without nutrition education and counselling has been associated with weight and height gain, increased linear growth, and decreased incidence of respiratory infections especially in food insecure populations [12,13,14].

**Figure 1:**
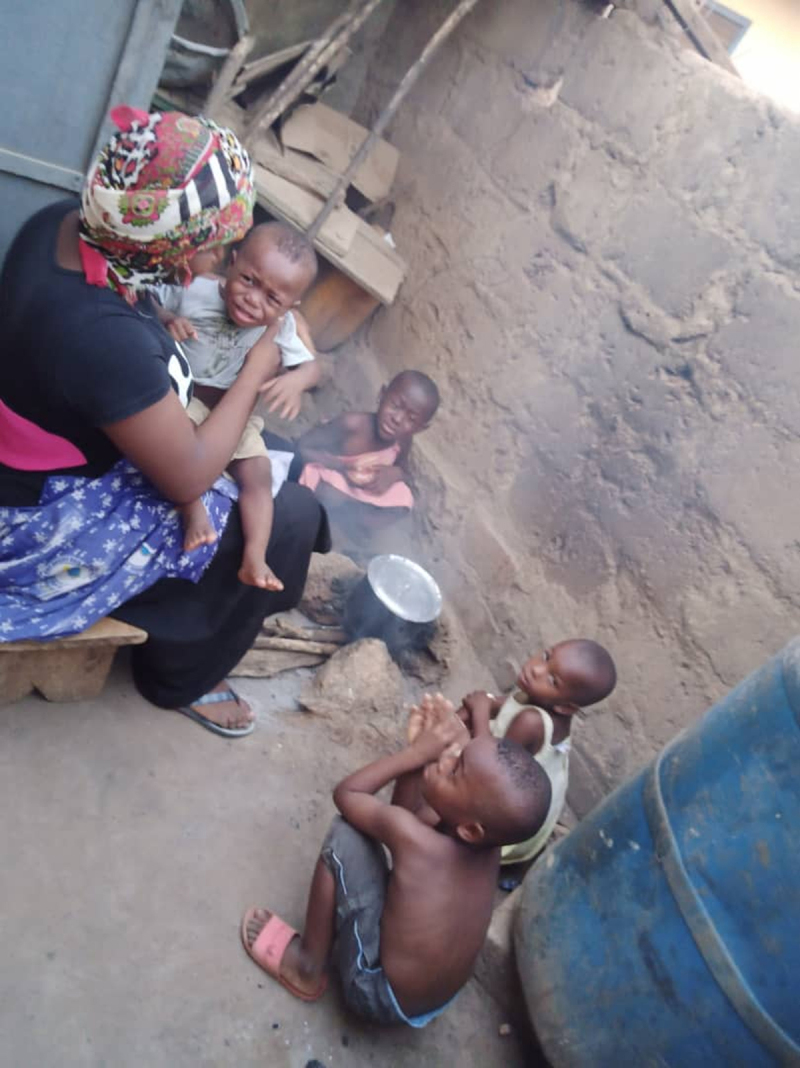
A woman cooking with wood and surrounded by her children.

Household air pollution from solid fuel use has been associated with risk of childhood pneumonia, acute lower respiratory infections, stunting, underweight, childhood anemia, and neonatal and child mortality [15,16,17]. In SSA and other developing regions where solid fuels are predominantly used, women are customarily responsible for cooking and very often, are accompanied by their young children whilst cooking. Both are exposed to the resulting air pollution as a result with the scenario depicted in Figure 1.

Poor water, sanitation and hygiene (WASH) conditions have detrimental effect on child growth and development owing to continual exposure to enteric pathogens, and associated wider social and economic complexities [18]. There is an increased risk of diarrheal diseases among children who do not wash their hands [19]. Access to improved water and sanitation is associated with reduced risk of diarrhea illnesses, stunting, and child mortality [20]. Repeated bouts of diarrhea cumulatively increases the risk of childhood stunting [21]. A multi-country ecological study also found improvement in population water and sanitation access to significantly decrease infant and under-five mortality [22].

## Way Forward

### Strengthening Child Welfare Clinics through Digital Technologies

In many developing countries, community health workers (CHW) are responsible for provision of health services in rural and remote communities. This is because primary healthcare centers are usually several kilometers away from these communities thereby making access a challenge for residents of these communities. CHWs in these countries are trained to administer a compendium of care with provision of nutrition-specific advice a key component of the care. CHWs, however, have limited training in nutrition which hampers the nutrition advice they are expected to provide to enhance child growth and development.

Smartphone technology should be leverage to improve child welfare services through the development of mobile applications for effective triage of nutrition care and provision of accurate, reliable, consistent, timely and up to date nutrition advice tailored toward the needs of children. The mobile applications can also be used to advise mothers and caregivers during the triage about environmental conditions that adversely impacts child health and survival. The mobile applications can also be used to send short SMS messages on nutrition and environmental health tips in the dominant language of the child welfare service coverage area to mothers and caregivers on regular basis. A study assessing the feasibility of mobile health technology (mHealth) for the provision of maternal and child health services in a deprived region of Ghana found the intervention to have the potential of eliminating barriers to equitable access to maternal and child healthcare services in rural areas [23]. A study evaluating the effectiveness of Rwanda’s nationwide mHealth programme reported that, with adequate resources, the programme can increase use of maternal and child health services [24]. Barriers to digital technology for improving child health and survival include low mobile network penetration, limited or erratic power supply, and poor mobile network connectivity in many countries. However, in many SSA countries mobile network penetration stands at more 90%.

### Investment in School Feeding Programmes (SFP)

There is compelling evidence on the benefits of SFPs for child health and nutrition [25,26,27]. Well-designed and properly implemented SFPs assures nutrient adequacy of children leading to improved nutritional status, decreased morbidity, and increased cognitive abilities. SFPs also drives children into school and keep them there, guarding against their stay at home and subsequent use by parents and relatives for water and fuel fetching, agriculture activities, and vending and other economic activities that threatens their health and survival. However, countries need to guard against politicization of the programme, the main challenge impeding effectiveness and threatening sustainability of SFPs in the limited SSA countries where they have been implemented. In these countries, programme managers at the various levels of operation and caterers assigned to schools earn their positions because of their political leanings and as a result, are replaced whenever the government changes. Ghana is a typical example. Programme managers and school caterers should earn their positions on merit and be given performance contracts. That way, nutritional quality of the meals served which has always been questioned in the countries where the programme is politicized will improve tremendously as managers and caterers will be accountable.

### Addressing Household Air Pollution (HAP)

There is limited evidence from randomized cookstove intervention trials conducted in Ghana, Nigeria, Malawi, and Nepal on the benefits for child health and survival of the use of liquefied petroleum gas (LPG), ethanol and improved cookstove for cooking [28,29,30,31]. Major barriers to the adoption of LPG and other clean cooking solutions in developing countries are poverty and supply chain issues. To overcome the poverty barrier, governments should heavily subsidize LPG and other clean cooking solutions, and also consider their provision as part of social protection programmes. To overcome the supply chain issues, governments should create an enabling environment for private sector involvement in LPG production and distribution, and innovations in clean cooking technologies.

Another source of HAP in SSA countries is the open burning of solid waste in households owing to limited and irregular waste collection services in many neighbourhoods.

Guarding against open burning of waste in households also demands private sector involvement to ease the burden on municipal authorities, as well as ensuring universal waste collection and curtailing the proliferation of informal dumpsites.

Creating awareness on the dangers of solid fuel use and open burning of waste through community durbars and religious fellowships could also help to address HAP. Governments and their development partners should support local non-governmental organizations and community-based organizations to take up the task.

### Improving Water, Sanitation and Hygiene (WASH) Services in Basic Schools

WASH remains an essential intervention for improved child health and development, and is very fundamental to sustainable development [32]. Improved WASH services prevents the two syndromes that are the common causes of child deaths globally; diarrhoea and acute lower respiratory infection [33]. Several WASH interventions have been associated with lower risk of diarrhoeal morbidity and will enable meeting the SDG targets [34,35]. Handwashing interventions have also been found reduce school absenteeism, respiratory tract infections, and laboratory confirmed influenza-like illness [36].

WASH content should be incorporated into the curriculum of basic schools at all levels to assure increased WASH knowledge of pupils for enhancing proper attitudes and practices. Ministries of Education should encourage the formation of health clubs in basic schools to provide a platform for promoting proper WASH attitudes and practices in schools and communities. Through meetings of the health club, the capacity of pupils in WASH promotion can be developed to enable them become effective change agents.

**Figure 2:**
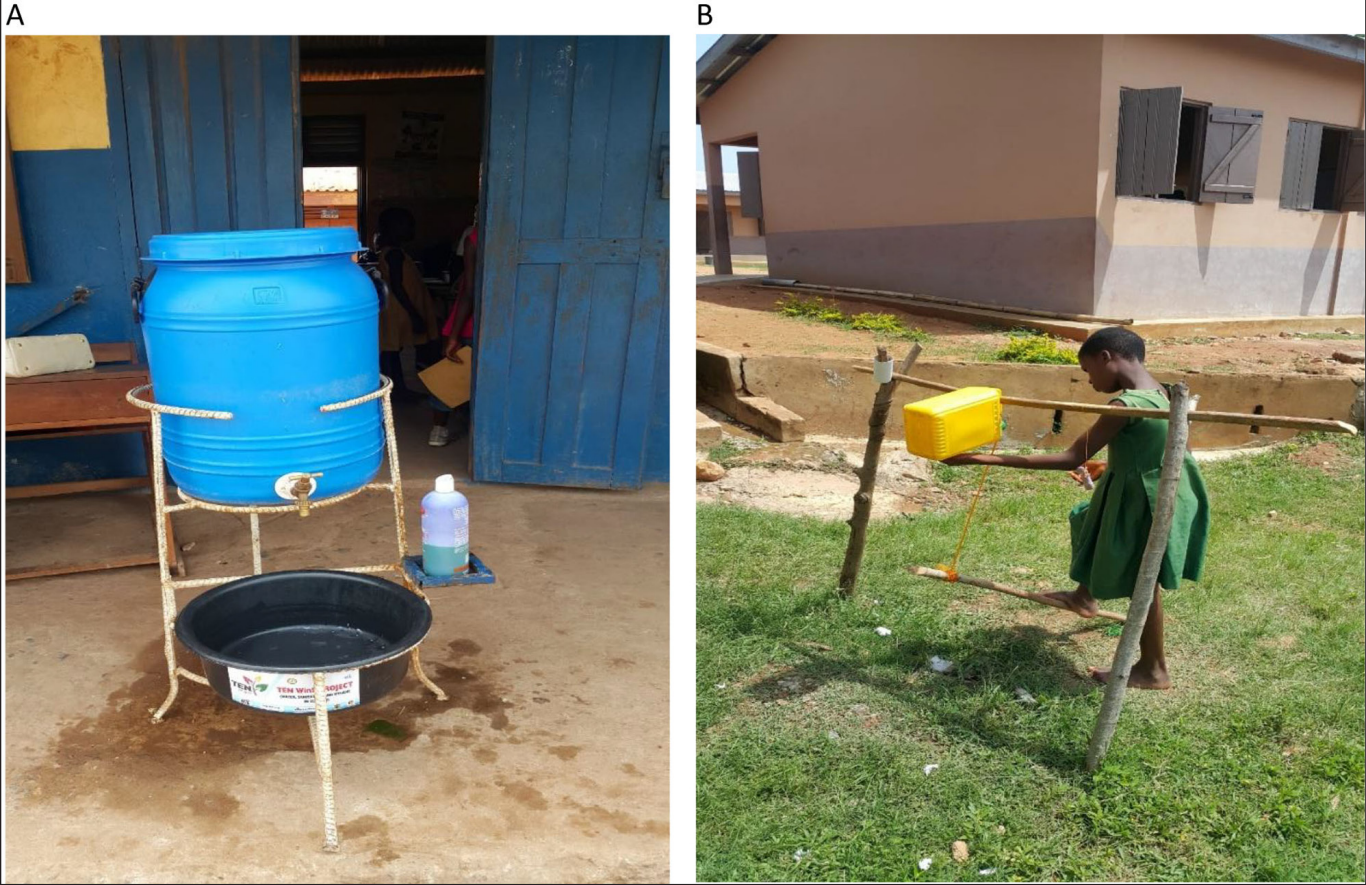
**A:** Veronica bucket; **B:** Pupil using tippy tap for handwashing.

Sustained use of veronica buckets (Figure 2A), a key infrastructure for promoting handwashing in schools in SSA countries, is threatened by the inability of many schools to maintain and/or replace them when they become damaged owing to financial constraints. Many schools are also unable to provide soap for handwashing for the same reason. Local governments should supply schools in their localities with veronica buckets and soap regularly to promote and sustain handwashing practices in schools. Tippy tap (Figure 2B), an improvisation of veronica buckets, and made from local raw materials is recommended in places where sustained use of veronica buckets is a challenge.

Local governments should also invest in the construction of toilets facilities in schools and provide access to improve water sources through the construction of mechanized boreholes and payment of water utility bills of schools.

## Conclusion

In conclusion, improving child health and survival in Sub-Saharan Africa and achieving the SDG targets will require a package of interventions beyond health system strengthening. There is ample empirical evidence on the effectiveness of the proposed interventions. Governments should therefore invest in these interventions by resourcing and strengthening the appropriate ministries, departments and agencies to act. There are barriers to effective implementation of the proposed interventions in countries but are surmountable with strong political will and involvement of the private sector.
